# Utility of Quantitative ^99m^Tc-MAA SPECT/CT for ^90^yttrium-Labelled Microsphere Treatment Planning: Calculating Vascularized Hepatic Volume and Dosimetric Approach

**DOI:** 10.1155/2011/398051

**Published:** 2011-07-28

**Authors:** Etienne Garin, Yan Rolland, Laurence Lenoir, Marc Pracht, Habiba Mesbah, Philippe Porée, Sophie Laffont, Bruno Clement, Jean-Luc Raoul, Eveline Boucher

**Affiliations:** ^1^Department of Nuclear Medicine, Comprehensive Cancer Center Eugène Marquis, CS 44229, 35042 Rennes, France; ^2^Faculty of Medicine, European University of Brittany, 35043 Rennes, France; ^3^INSERM U991 Liver Metabolisms and Cancer, 35033 Rennes, France; ^4^Department of Medical Imaging, Comprehensive Cancer Center Eugène Marquis, CS 44229, 35042 Rennes, France; ^5^Department of Medical Oncology, Comprehensive Cancer Center Eugène Marquis, CS 44229, 35042 Rennes, France; ^6^Department of Medical Informatics, Comprehensive Cancer Center Eugène Marquis, CS 44229, 35042 Rennes, France

## Abstract

*Objectives*. The aim of this study was to assess the effectiveness of SPECT/CT for volume measurements and to report a case illustrating the major impact of SPECT/CT in calculating the vascularized liver volume and dosimetry prior to injecting radiolabelled yttrium-90 microspheres (Therasphere). *Materials and Methods*. This was a phantom study, involving volume measurements carried out by two operators using SPECT and SPECT/CT images. The percentage of error for each method was calculated, and interobserver reproducibility was evaluated. A treatment using Therasphere was planned in a patient with three hepatic arteries, and the quantitative analysis of SPECT/CT for this patient is provided. *Results*. SPECT/CT volume measurements proved to be accurate (mean error <6% for volumes ≥16 cm^3^) and reproductive (interobserver agreement = 0.9). In the case report, ^99m^Tc-MAA SPECT/CT identified a large liver volume, not previously identified with angiography, which was shown to be vascularized after selective MAA injection into an arterial branch, resulting in a large modification in the activity of Therasphere used. *Conclusions*. MAA SPECT/CT is accurate for vascularized liver volume measurements, providing a valuable contribution to the therapeutic planning of patients with complex hepatic vascularization.

## 1. Introduction

Hepatocellular carcinoma (HCC) is a common cancer, and its treatment is difficult. A locoregional treatment may be proposed to certain patients, involving either chemoembolization or radioembolization. For radioembolization, ^131^I-lipiodol has been used for many years with good results [1], but its use is limited by major radioprotection problems.

Recently, radioembolization using microspheres labeled with yttrium-90 has been developed, notably TheraSphere (MDS Nordion, Ottawa, Canada) [2–6]. This approach is based on delivering a high radioactive dose to the liver volume to be treated. When both lobes are affected, two treatments must be carried out at 1-month interval in order to minimize the risk of radiation-induced side effects [4]. The treatment requires a first diagnostic angiography, with the aim of coiling collateral gastrointestinal vessels and performing an MAA scintigraphy in order to obtain a perfusion hepatic scintiscan and calculate the percentage of pulmonary shunt.

The activity to be injected (*A*_inj_) is calculated based on a well-defined model. The aim is to deliver a dose *D*_(Gy)_ of 120 ± 20 Gy to the volume to be treated. This dose is calculated according to the following formula that is based on the previously described MIRD formalism [5] and widely used:

(1) $$D_{\left(\text{Gy}\right)}=\frac{A_{\text{inj}(\text{GBq})}\cdot \left(1-S\right)\cdot 50}{M_{\left(\text{Kg}\right)}},$$

where *S* represents the percentage of pulmonary shunt and *M* the mass of vascularized hepatic volume, with the mass of volume to be treated corresponding to the volume multiplied by a correction factor of 1.03 [5]. This volume corresponds to the volume of the hepatic parenchyma vascularized by the hepatic artery into which the microspheres are injected. Generally, the calculation is based on CT. 

A precise calculation of the vascularized volume is essential for dosimetric calculation, including the activity to be administered.

The vascularized or functional liver volume may also be analyzed using a vascular tracer such as human albumin serum labeled with technetium-99m (^99m^Tc-MAA): the volume of distribution of ^99m^Tc-MAA following selective injection into the hepatic artery, at the same position where the microspheres are to be injected, reflects the vascularized volume of the lobe to be treated. 

In the absence of anatomical vascular variations, the definition of the vascularized liver volume is relatively easy to assess using CT. Contrarily, this calculation is much more problematic in the event of anatomical variations, such as when three distinct arterial branches vascularize the liver, a situation encountered in about 10% to 30% of patients [6]. It is assumed that the right vascular branch vascularizes segments 6 and 8, the central branch segment 5 and 7, the left branch segments 2, 3, and 4, while the inconsistent vascularization of segment 1 is widely recognized. Given this case scenario, MAA scintigraphy, which is mandatory for evaluating lung shunting, may be used for calculating the functional liver volume vascularized by each arterial branch.

SPECT has already been employed for calculating functional volumes [7, 8] although it may lead in certain cases to volume overestimation due to the thresholding method used. In addition, PET has been reported to be useful for functional volume measurements, in particular in defining the volumes of irradiation [9–11]. The method used for volume definition (simple visual method or based on an isocontour with predefined threshold) was shown to significantly impact on the achieved values [10]. 

While the use of SPECT/CT has not yet been reported in this setting, SPECT/CT-fused images may be helpful in delineating volumes, which may prove to be a reliable measurement of functional volumes. SPECT/CT may also be used to measure the tumor volume and nontumoral injected liver volume in order to calculate the doses absorbed by the tumor and the nontumoral liver, respectively.

In this paper, we report on a phantom study aimed at validating volume measurements based on SPECT/CT. We also stress the advantages of SPECT/CT in computing the vascularized liver volume in addition to calculating the doses absorbed by the healthy liver and tumor in a patient with complex hepatic vascularization.

## 2. Materials and Methods

### 2.1. Phantom Study

We carried out a study on phantoms in order to validate the volume measurements performed using SPECT/CT. Various phantoms were used, including a cylindrical Jaszack phantom with a volume of 6,716 mL (cylinder 1) and two cylindrical phantoms of 774 (cylinder 2) and 473 mL (cylinder 3) for the measurement of large volumes (mimicking the liver). Spheres 1, 2, 3, and 4 of 55, 20.5, 16, and 8 mL, respectively, were used and inserted into the Jaszack phantom for measuring small representative tumor volumes. The activities of ^99m^Tc used for phantoms representative of the liver (cylinder 1, 2, and 3) ranged from 55 to 170 MBq. For the spheres, activities of 18.5, 37, 55.5, and 74 MBq were used. These activities were chosen in order to simulate standard clinical situations involving an injection of 185 MBq of ^99m^Tc-MAA in a liver of 1500 mL, with tumor uptake of 10%, 20%, 30%, and 40% of the injected activities, respectively. 

SPECT/CT acquisitions were performed (32 projections, 180°, 128∗128, 30 s/projection, Symbia T2 gantry, Siemens). SPECT imaging data was reconstructed using an iterative method (OSEM) with attenuation and scatter correction, and images were then visualized with or without fusion with CT scan data.

Volume measurement was carried out on SPECT and SPECT/CT images using a Syngo data-processing console display unit (Siemens) with “Volume Analysis” software. This software allowed us to generate semi-automatically the volume-of-interest (VOI) in the liver and tumor by means of an isocontour definition method. Each voxel with an activity reaching or exceeding a threshold percentage of the highest activity was included in the VOI. 

**Figure 1:**
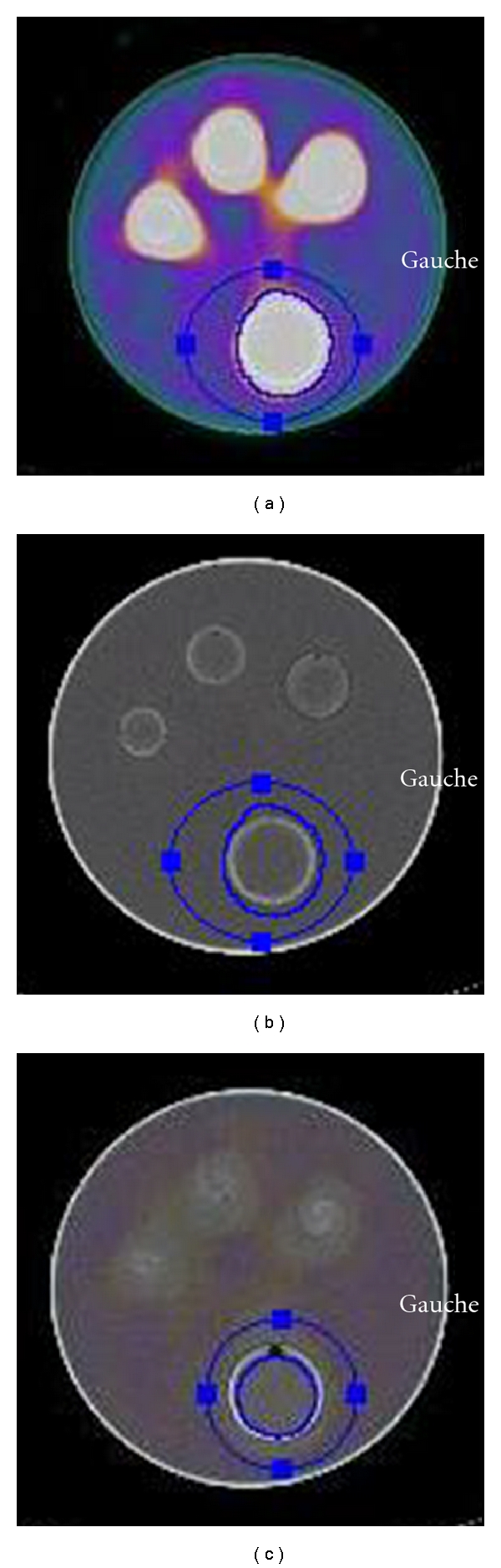
Delineation of VOIs used for quantitative analysis of SPECT and SPECT/CT analysis (a, b): VOI defined on SPECT hot spot alone. Fused SPECT/CT image with the VOI matching with the hot spot ((a), SPECT/CT scale); CT scale showing that the VOI is not accurate and larger than the sphere (b). (c): VOI defined with SPECT/CT and using the CT scale: the isocontour was fitted by superposition on the boundaries of the internal wall of the sphere.

For each volume measurement, the isocontour was fitted by superposition on either the contour of the hot spot located by SPECT alone or the internal wall of the phantom located on the SPECT/CT fusion images (Figure 1). 

Measurements of volumes by SPECT and SPECT/CT were carried out by two operators blinded to the phantom volumes.

### 2.2. Statistical Analysis

For each method, the percentage of error was calculated by reference to the actual volume of the phantoms. The interobserver reproducibility was evaluated using the Bland-Altman test of agreement, with values ≥0.8 considered to be excellent, values in the range of 0.6–0.8 to be good, values in the range of 0.4–0.6 to be poor, and values <0.4 to be very poor.

### 2.3. Case Report and Dosimetric Aproach

**Figure 2:**
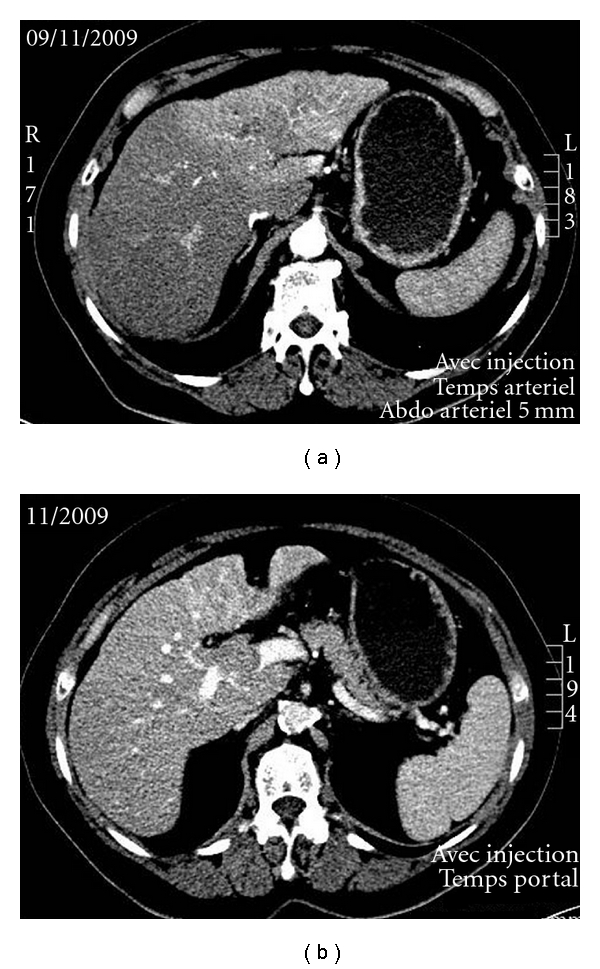
case report: patient with a large HCC Contrast enhanced CT evidencing a large infiltrative tumor of the left liver (a) with a portal vein thrombosis (b).

We report on the case of a 63-year-old patient with a voluminous HCC infiltrating the whole left liver (Figure 2) along with portal vein thrombosis, who was addressed to our center for radioembolization with 90Y-loaded glass microspheres. 

**Figure 3:**
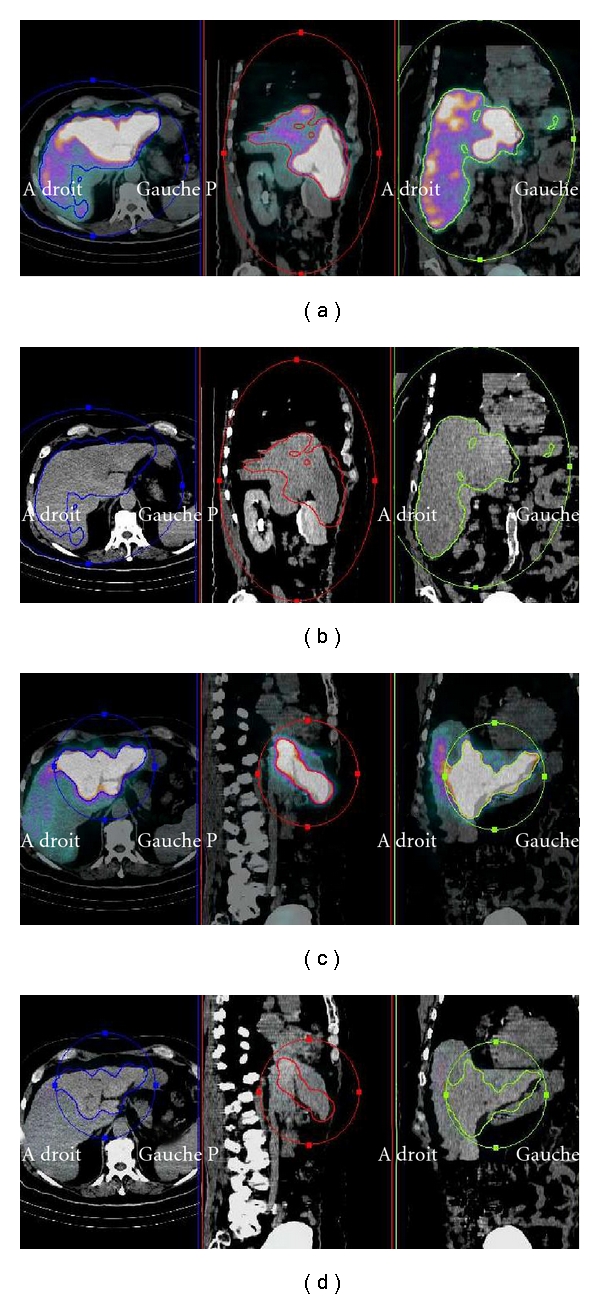
Case report: delineation of the tumoral and injected lover VOI on SPECT/CT whole; injected liver VOI with SPECT/CT color scale (a), whole injected liver VOI with CT scale and visualisation of SPECT isocontour (b), tumoral VOI with SPECT/CT color scale (c) and tumoral VOI with CT scale and visualisation of SPECT isocontour (d).

At the end of the diagnostic angiography, 185 MBq of ^99m^Tc-MAA was injected selectively for SPECT/CT. The parameters of acquisition and reconstruction were the same as those used for the phantom study. SPECT/CT quantitative analysis (volume and count measurements) was conducted on the fusion images. For each VOI, the threshold value was adjusted so that the isocontours of the distribution volume of MAA were superimposed on the fusion images that corresponded to the contours of the liver and tumor (Figure 3). These VOIs were then used to measure the distribution volume of ^99m^Tc-MAA in the liver and tumor (expressed in mL) in addition to the total activity (expressed in counts) contained in the liver (CP_L_) and tumor (CP_tum_). Volume and total counts in the healthy liver (CP_HL_) were calculated by subtracting liver and tumor parameters. 

The dose absorbed by the tumor was calculated based on the standard formula as follows:

(2) $$D_{\text{tum}(\text{Gy})}=\frac{A_{\text{tum}(\text{GBq})}\cdot 50}{W_{\text{tum}(\text{Kg})}},$$

where *A*_tum_ = activity contained in the tumor expressed in GBq, *A*_tum_ = (*A*_inj(GBq)_ · (1 − *S*) · CP_tum_)/((CP_tum_ + CP_HL_)), *W*_tum_ = mass of tumor.

The dose absorbed by the healthy injected liver was calculated based on the standard formula as follows:

(3) $$D_{\text{HL}(\text{Gy})}=\frac{A_{\text{HL}(\text{GBq})}\cdot 50}{W_{\text{HL}(\text{kg})}},$$

where *A*_HL_ = activity contained in the healthy liver expressed in GBq, *A*_HL_ = (*A*_inj(GBq)_ · (1 − *S*) · CP_HL_)/((CP_HL_ + CP_tum_)), *W*_HL_ = mass of healthy liver.

The Therasphere injection was administered 8 days later during the second angiography, with the aim to administer a dose of 120 ± 20 Gy to the vascularized volume. 

## 3. Results

### 3.1. Phantom Study

In total, acquisitions and measurements were carried out on 23 test objects of different configuration (size and activity), with results for both operators provided in Tables 1 and 2.

For SPECT alone, mean errors of volume measurements were relatively high (>20%, Table 3), which was due to over- or underestimation of volumes by observer 1 and, more frequently, to large volume overestimation by observer 2.

Interobserver reproducibility was inadequate with SPECT alone, as the Bland-Altman test result was only 0.2.

For SPECT/CT, results were superior, as mean errors of measurement were <10% for the two operators, being <6% for the measurement of volumes ≥16 cm^3^ and even <2.5% for volumes ≥473 mL (Table 3). Interobserver reproducibility was good when using SPECT/CT, with a Bland-Altman test result of 0.9.

### 3.2. Case Report and Dosimetric Approach

**Figure 4:**
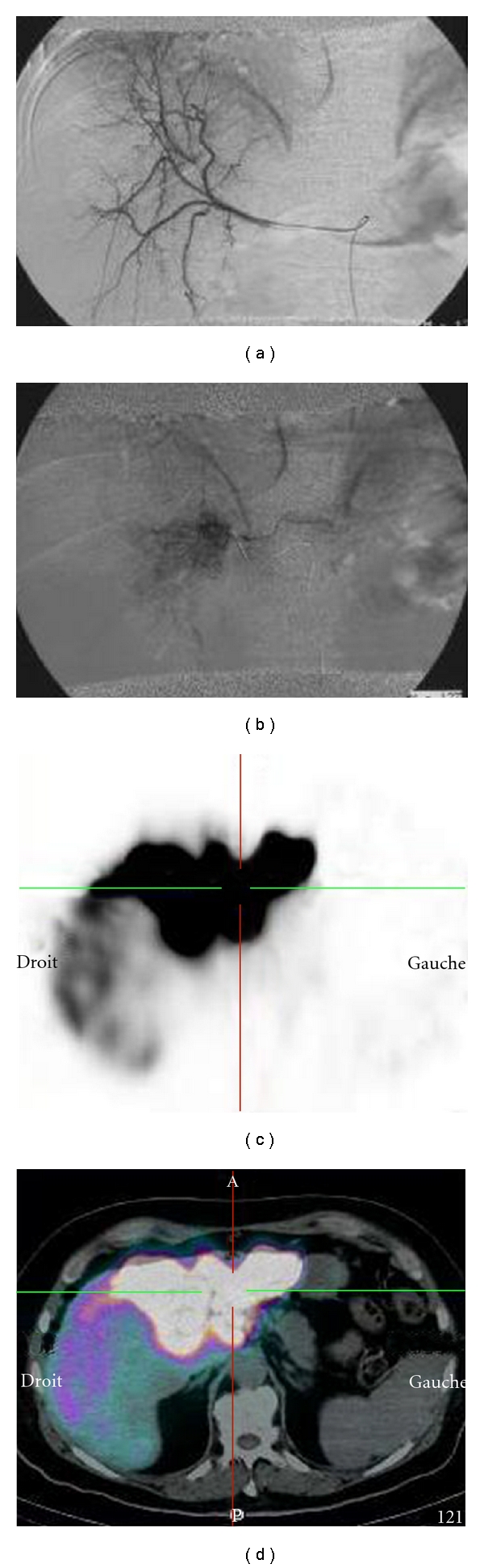
Case report: angiographic and scintigraphic data; right selective angiography (a), selective angiography of the common hepatic artery vascularizing segment IV and the left liver (b), SPECT (c) and SPECT/CT (d) after injection of the MAA at the level of the central hepatic artery revealing an unexpected uptake in the right liver, in addition to the expected uptake of segment IV and left liver (whole liver distribution, volume 1829cc).

Diagnostic angiography revealed an anatomical variant with three arterial branches vascularizing the liver: a right hepatic artery originating from the mesenteric artery (Figure 4(a)), and a common hepatic artery from the celiac trunk leading to a central branch and left branch vascularizing the central and left liver, respectively (probably segments 2, 3, and 4 but not the whole liver; see Figure 4(b)).

Unexpectedly, after injecting MAA into the common hepatic branch, SPECT/CT revealed the whole liver distribution of the MAA (Figure 4(c)).

After injecting contrast or MAA into the common hepatic branch, the vascularized liver volume was only 346 mL based on angiographic + CT data versus 1829 mL with quantitative MAA SPECT. Moreover, the tumoral uptake calculated using quantitative MAA SPECT data represented 69.1% of the total liver uptake with no lung shunting apparent.

Based on angiographic + CT, the activity of ^90^Y-loaded microspheres to be injected in order to obtain a dose of 120 Gy in the injected volume, presumed to be only the left liver, would have been 0.8 GBq. 

Based on quantitative MAA SPECT analysis, a 5 GBq activity was planned to be used, resulting in a dose of 132 Gy to the vascularized liver (whole liver), a tumoral dose of 275 Gy, and a nontumoral injected liver dose of only 57 Gy.

**Figure 5:**
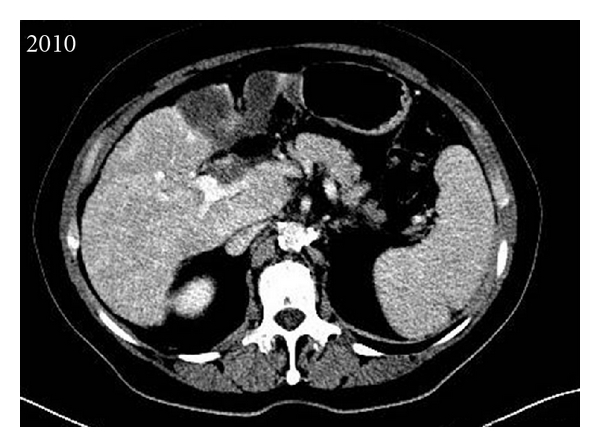
Case report: follow-up CT scan 4 months after injection of 5 GBq of in the central branch: major response.

Based on quantitative MAA SPECT/CT analysis instead of standard angiographic and CT data, the patient was treated with 5 GBq Therasphere. No toxicity was observed, and a major response was achieved (Figure 5). At the last follow-up visit, 14 months after treatment, the patient showed no evidence of recurrence.

## 4. Discussion

SPECT is currently used to define functional volumes of different organs such as the liver [7, 8], and the difficulties relating to the thresholding methods are widely recognized. This is particularly true in the case of small volumes and organs with low contrast (organ/background ratio <5) [12]. Data pertaining to SPECT for the calculation of tumoral volume is scare. Tumoral volume measurements have been extensively reported and are still being performed using PET for defining the volumes of irradiation [9–11]. However, due to the variability in the thresholding methods resulting in widely variable data, determining tumoral volumes based on PET images is still challenging [9]. For example, using different approaches for volume definition (visual evaluation, isocontour with a 40% threshold, isocontour with a 2.5 SUV threshold, and adaptive tumor/background ratio threshold) Lee [10] showed that volumes generated were highly dependent on the method used with intermethod variability exceeding 100%.

We describe a new method of organ and tumor volume calculation based on SPECT/CT data using simple manufactured software. This adaptive thresholding method was based on a direct visualization of tumoral and liver boundaries on SPECT/CT images, avoiding the use of thresholds dependent on predefined values. We showed that SPECT alone (visual delimitation of the volume based on the hot spot) was acceptable for large volumes (error <10% for volumes >473 mL), but inaccurate for small volume measurements, in line with previously published literature. In fact, visual volume definition is highly dependent on the individual investigator and display window setting (type of grey or color window and saturation used), potentially leading to large volume over- or underestimation [10]. With the SPECT/CT quantitative method used in our study, the anatomical information supplied by CT allows for VOI guidance so that the definition of volumes is not only based on the visual adjustment of VOI on uptake, but also on the anatomical outlines of both liver and tumor. This approach turned out to be highly accurate, as the error of measurement was <6% for volumes larger than 16 mL and even <2.5% for very large volumes, such as the liver, with excellent interobserver agreement. 

The case reported in our study clearly demonstrates that in patients with anatomical abnormalities in liver vascularization, MAA SPECT/CT vascularized volume measurement is a more functional and reliable method than volume calculations using the anatomical Couinaud segmentation based on angiographic and CT data. In our case report, due to MAA SPECT/CT, we were able to detect an unexpectedly large volume of liver slightly vascularized after selective injection of MAA into the common hepatic branch. This method revealed the existence of microvascular communications between different anatomic segments, probably via intratumoral arterioportal shunts with low arterial blood flow, which were not visible on angiography but detected using MAA SPECT/CT.

The therapeutic impact for our patient was crucial, since it led us to significantly increase the activity injected for treatment, 5 GBq instead of 0.8 GBq, without any toxicity but an excellent response.

Furthermore, the evaluation of tumor and healthy liver doses based on the quantitative analysis of SPECT/CT is of great interest. In fact, the dose absorbed by the tumor represents the parameter most likely to correlate with response. This parameter depends directly on the quantity of radioactivity fixed in the tumor (i.e., degree of vascularization) as well as the total quantity of microspheres injected. Only MAA SPECT/CT allows us to evaluate this parameter, which corresponds to the tumoral absorbed dose.

## 5. Conclusion

The effectiveness and reproducibility of MAA SPECT/CT volume measurements was confirmed by a phantom study, with a mean error <6% for volumes ≥16 mL and <2.5% for larger volumes, such as the whole liver. MAA SPECT/CT appears to be a more functional tool in identifying and calculating vascularized liver volumes than angiography, as it is able to identify unexpected vascularized areas with low blood flow not recognizable by angiography. In addition, quantitative MAA SPECT/CT allows for calculating the tumoral absorbed and nontumoral injected liver doses. Therefore, quantitative MAA SPECT/CT may be of great help in defining vascularized liver volumes and calculating the activity to be administered, especially in patients with complex hepatic vascularization. Quantitative MAA SPECT/CT may be instrumental in optimizing the activity to be injected, thereby increasing the therapeutic effectiveness. Nonetheless, this hypothesis must still be validated in controlled studies.

## Figures and Tables

**Table 1:** Operator 1 results.

Phantom configuration		True sphere/ background ratio	SPECT alone			SPECT- CT		
			Isocontour (%)	Measured volume (cm^3^)	Error (%)	Isocontour (%)	Measured volume (cm^3^)	Error Vol. (%)
18,5 MBq/sphere	Cylinder 1	—	2%	6521	−2,90	2%	6521	−2,90
	Sphere 1	2.8	42%	75,45	37,18	48%	57,22	4,04
	Sphere 2	7.6	31%	22,35	6,43	27%	27,08	28,95
	Sphere 3	9.6	27%	18	12,50	29%	16,94	5,88
	Sphere 4	19.6	15%	14,72	84,00	19%	10,91	36,38

37 MBq/sphere	Cylinder 1	—	2%	6180	−7,98	1%	6735	0,28
	Sphere 1	6	46%	44,86	−18,44	42%	49,06	−10,80
	Sphere 2	17	25%	22,58	7,52	29%	20,52	−2,29
	Sphere 3	23.4	23%	17,55	9,69	27%	14,95	−6,56
	Sphere 4	43.8	31%	10,45	30,63	35%	9,08	13,50

55,5 MBq/sphere	Cylinder 1	—	1%	6737	0,31	1%	6737	0,31
	Sphere 1	9.9	44%	45,32	−17,60	31%	59,28	7,78
	Sphere 2	29.6	33%	18,62	−11,33	31%	19,3	−8,10
	Sphere 3	38.6	33%	17,32	8,25	35%	16,17	1,06
	Sphere 4	73.1	31%	14,37	79,63	40%	10,53	31,63

74 MBq/sphere	Cylinder 1	—	NA	NA	NA	NA	NA	NA
	Sphere 1	16	52%	33,72	−38,69	31%	54,86	−0,25
	Sphere 2	45	21%	23,5	11,90	27%	19,81	−5,67
	Sphere 3	57	21%	18,05	12,81	27%	15,49	−3,19
	Sphere 4	114	31%	5,95	−25,63	21%	8,32	4,00

55 MBq	Cylinder 2	—	46%	709	−8,40	25%	795	2,71
116 MBq	Cylinder 2	—	46%	667	−13,82	35%	770	−0,52
72 MBq	Cylinder 3	—	33%	455	−3,81	23%	515	8,88

**Table 2:** Opertor 2 results.

Phantom configuration		True sphere/ background ratio	SPECT alone			SPECT-CT		
			Isocontour (%)	Measured volume (cm^3^)	Error (%)	Isocontour (%)	Measured volume (cm^3^)	Error (%)
18,5 MBq/sphere	Cylinder 1	—	1%	7087	5,52	2%	6521	−2,90
	Sphere 1	2.8	31%	207	276,36	48%	57,2	4,00
	Sphere 2	7.6	13%	104,5	397,62	28%	24,4	16,19
	Sphere 3	9.6	19%	27,4	71,25	29%	16,9	5,62
	Sphere 4	19.6	8%	29,2	265,00	19%	10,9	36,25

37 MBq/sphere	Cylinder 1	—	2%	6180	−7,98	1%	6700	−0,24
	Sphere 1	6	7%	169,7	208,55	34%	53,56	−2,62
	Sphere 2	17	3%	113,7	441,43	23%	24,34	15,90
	Sphere 3	23.4	3%	119,78	648,63	27%	14,9	−6,88
	Sphere 4	43.8	4%	53,48	568,50	33%	9,8	22,50

55,5 MBq/sphere	Cylinder 1	—	1%	6737	0,31	1%	6737	0,31
	Sphere 1	9.9	10%	159,3	189,64	35%	55,7	1,27
	Sphere 2	29.6	4%	85	304,76	25%	22,7	8,10
	Sphere 3	38.6	7%	51,5	221,88	36%	15,5	−3,13
	Sphere 4	73.1	6%	44,1	451,25	36%	12	50,00

74 MBq/sphere	Cylinder 1	—	1%	6565	−2,10	1	6565	−2,10
	Sphere 1	16	15%	99,5	80,91	32%	56	1,82
	Sphere 2	45	6%	54,1	157,62	23%	23,7	12,86
	Sphere 3	57	5%	42,8	167,50	25%	17,4	8,75
	Sphere 4	114	2%	35,8	347,50	20%	8,7	8,75

55 MBq	Cylinder 2	—	17%	887	14,60	27%	801	3,49
116 MBq	Cylinder 2	—	19%	855	10,47	29%	775	0,13
72 MBq	Cylinder 3	—	21%	520	9,94	27%	481	1,69

**Table 3:** Mean error (±1 standard deviation) with SPECT and SPECT/CT volume measurements.

	SPECT alone			SPECT/CT		
	All volumes	volumes ≥16 mL	volumes ≥473 mL	All volumes	volumes ≥16 mL	volumes ≥473 mL
Operator 1	20.4 ± 22.4%	12.8 ± 10.3%	6.2 ± 4.8%	8.5 ± 10.5%	5.6 ± 6.7%	2.2 ± 3.1%
Operator 2	210.8 ± 194.5%	169.3 ± 181.3%	7.2 ± 4.9%	9.4 ± 12.3%	5.2 ± 5.1%	1.5 ± 1.3%
